# A Novel Sidelobe Reduction Algorithm Based on Two-Dimensional Sidelobe Correction Using D-SVA for Squint SAR Images

**DOI:** 10.3390/s18030783

**Published:** 2018-03-05

**Authors:** Min Liu, Zhou Li, Lu Liu

**Affiliations:** 1 Qian Xuesen Laboratory of Space Technology, Beijing 100094, China; liumin@qxslab.cn (M.L.); liulu@qxslab.cn (L.L.); 2 Beijing Institute of Remote Sensing Information, Beijing 100192, China

**Keywords:** squint SAR, sidelobe reduction, double spatially variant apodization (D-SVA), non-integer Nyquist sampled imagery, impulse response

## Abstract

Sidelobe reduction is a very primary task for synthetic aperture radar (SAR) images. Various methods have been proposed for broadside SAR, which can suppress the sidelobes effectively while maintaining high image resolution at the same time. Alternatively, squint SAR, especially highly squint SAR, has emerged as an important tool that provides more mobility and flexibility and has become a focus of recent research studies. One of the research challenges for squint SAR is how to resolve the severe range-azimuth coupling of echo signals. Unlike broadside SAR images, the range and azimuth sidelobes of the squint SAR images no longer locate on the principal axes with high probability. Thus the spatially variant apodization (SVA) filters could hardly get all the sidelobe information, and hence the sidelobe reduction process is not optimal. In this paper, we present an improved algorithm called double spatially variant apodization (D-SVA) for better sidelobe suppression. Satisfactory sidelobe reduction results are achieved with the proposed algorithm by comparing the squint SAR images to the broadside SAR images. Simulation results also demonstrate the reliability and efficiency of the proposed method.

## 1. Introduction

Synthetic aperture radar (SAR) transmits linear frequency modulation (LFM) signals and receives the backscattered echoes from Earth observation scenes. Typically, SAR images can be obtained with high spatial resolution by different image formation techniques when SAR works in broadside or slightly squint modes. However for some special applications, only squint mode SAR can satisfy such demands. In comparison, the squint SAR provides more mobility and flexibility. However, their severe echo signal range-azimuth coupling issues make squint SAR imaging algorithms more complicated than those for broadside SAR [1,2].

The impulse response of a point target in one dimension can be written as a sinc function (sin(*πx*)/(*πx*)) in the image domain [3]. For a strong scatterer, its sidelobe energy falls off gradually away from the mainlobe. If a weak scatterer happens to locate in the sidelobe regions of the strong scatterer, it can be masked or distorted easily when the sidelobe energy of the strong scatterer is much higher than the mainlobe energy of the weak scatterer. Therefore, it is very important to find an effective sidelobe reduction algorithm.

Various methods have been presented to control the sidelobe levels for SAR imagery [3,4,5,6,7,8,9]. Bidimensional linear apodization has been applied to data in frequency domain, which removes sidelobes for SAR images at the expense of some resolution degradation [3].

Alternatively, a spatially variant apodization (SVA) algorithm can suppress sidelobes and preserve image resolution at the same time [3,4,5,6,7,8,9]. Depending on the Nyquist sampling rate, SVA algorithm complexity might be very different. The simplest form is the three point convolution, which requires the data to be sampled at an integer multiple of the Nyquist frequency [3]. For those with sampling rate that is not the integer multiple of the Nyquist frequency, several modified algorithms have been proposed, but all at the expense of much larger computational complexity [5,6]. Up to present, most SVA algorithms are limited to broadside SAR images. Because the sidelobes of squint SAR images may not locate on the image principal axes (rows and columns), bi-dimensional SVA filters can hardly fully extract sidelobes information [9]. Hence for squint SAR images, such algorithms could obtain poor sidelobe reduction results. To address this issue, Castillo-Rubio et al. proposed an SVA algorithm for squint SAR images [9], in which the authors implemented the range migration algorithm (RMA) for image formation. By taking advantages of sampling parameters recalculation and nearest interpolation computation, the authors were able to extract most of the sidelobe information for the squint SAR images. However, this algorithm is complex and time consuming as it used 5-tabs SVA methods.

In this paper, we propose a novel sidelobe reduction algorithm using double spatially variant apodization (D-SVA) for squint SAR images. The first step is to pre-process the squint SAR images by aligning the sidelobe directions parallel or vertical to the principal axes (row and column). Then, the sidelobe suppression is achieved by applying the D-SVA algorithm to the pre-processed data. Finally, an inverse transform is performed as a post-processing for the squint SAR images. Simulation results demonstrate that this algorithm is reliable and efficient.

## 2. Sidelobe Control Algorithms for SAR Images

### 2.1. Traditional Linear Windowing

There are many classic windowing functions to suppress the impulse response (IPR) sidelobes for SAR images, such as Hamming, Taylor, Kaiser, and Blackman. Among all these windows, Hanning (cosine-on-pedestal) window is widely used to achieve low sidelobes, though at the cost of doubling the mainlobe width compared to the uniform (unweighted) signal data. Linear windows have the undesired side effect of broadening the IPR mainlobe width, which decreases the image resolution. If there is a high demand for SAR image resolution, linear windows could not meet the requirement to suppress sidelobe level whiling maintaining high image resolution at the same time.

### 2.2. Nonlinear Apodization (Windowing)

Nonlinear apodization (windowing) methods include spatially variant apodization (SVA), adaptive sidelobe reduction (ASR) [10], parametric windows, adaptive Kaiser window [11], etc. The most commonly used method is SVA or improved (modified) SVA, which applies a particular signal-domain windowing function to filter each pixel in SAR images. Whether the pixel is the mainlobe or sidelobe depends on the properties of the neighboring image pixels. Nonlinear apodization methods can keep high image resolution and suppress sidelobes at the same time. Furthermore, several super-resolution/bandwidth-extrapolation algorithms are presented based on Super-SVA [12,13,14].

### 2.3. Dual-Delta Factorization

Dual-delta factorization algorithm is performed based on the point spread function (PSF), which divides the PSF into a group of different simple subsystems (dual-Delta operators) [8]. It then factorizes the PSF as a group of dual-Delta operators and passes the observed signal through their inverses. The greedy strategy is used to suppress sidelobes in this algorithm.

### 2.4. Iterative Adaptive Approach (IAA)

IAA is an estimation algorithm based on iterative adaptive approach, which estimates the target’s backscatter coefficient to achieve low sidelobe levels for SAR images [15]. IAA is not a post-processing algorithm, and it can also be viewed as an imaging algorithm. First, the transition matrix for every pixel’s echo is built and the covariance matrix is calculated, and then the process is iteratively applied to calculate all the points’ backscatter coefficient. This approach gets low sidelobe levels by constant iterations and updates, which is rather complicated and time-consuming. The major merit for this algorithm is that it can recover weak targets.

## 3. Non-Integer Nyquist SVA Algorithm

SVA is a very effective technique to reduce the sidelobes of a SAR image sampled at an integer multiple of the Nyquist frequency. When processing the image with non-integer sampling rate by SVA algorithm, the sidelobes can not be completely suppressed [4]. In order to suppress sidelobes effectively of SAR image sampled at any case of Nyquist sampling rate, the filter is extended from 3-taps to 5-taps [6]. However, these algorithms rely on constrained optimizations, which introduced substantial computational cost.

The frequency domain weighting function can be written as:

(1) $$W(f)=a+2\omega_{1}\cdot \cos(2\pi l\frac{f}{f_{s}})$$

where *f_s_* is the sampling frequency, *f* is the frequency whose support region is $\left[-\frac{f_{0}}{2},\frac{f_{0}}{2}\right],\;f_{0}$ is the signal bandwidth, *a* is a constraint parameter which guarantees the unit gain at the center of the aperture, and *ω*_1_ is the parameter which can vary according to the weighting function.

The corresponding IPR of Equation (1) can be expressed as:

(2) $$w\left(m\right)=a\delta \left(m\right)+\omega_{1}\cdot \left[\delta \left(m-l\right)+\delta \left(m+l\right)\right]$$

(3) $$\delta \left(m\right)=sinc\left(w_{s}m\right)=\frac{\sin\left(\pi w_{s}m\right)}{\pi w_{s}m}$$

where *w*(*m*) is the Fourier transform (FT) of *W*(*f*), *m* is a sample point in discrete domain, $l=\frac{f_{s}}{f_{0}}$ (when *l* is a positive integer, the Nyquist sampling rate is integer. Otherwise, the Nyquist sampling rate is non-integer), $w_{s}=\frac{f_{0}}{f_{s}}$ represents the oversampling of the signal. Typically, SAR images are oversampled at non-integer Nyquist sampling rate. Therefore, in this section only the images with non-integer Nyquist sampling rate are considered.

Therefore, Equation (2) can be rewritten as:

(4) $$w(m)=a\cdot sinc(w_{s}m)+\omega_{1}\{sinc\left[w_{s}\left(m-\frac{f_{s}}{f_{0}}\right)\right]+sinc\left[w_{s}\left(m+\frac{f_{s}}{f_{0}}\right)\right]\}$$

The sampling points for SAR and ISAR images must be integers with non-integer Nyquist sampling rate. So Equation (4) can be rewritten as:

(5) $$I(m)=a\cdot sinc(w_{s}m)+\omega_{1}\{sinc\left[w_{s}\left(m-\left\lfloor \frac{f_{s}}{f_{0}}\right\rfloor \right)\right]+sinc\left[w_{s}\left(m+\left\lfloor \frac{f_{s}}{f_{0}}\right\rfloor \right)\right]\}$$

where the sign ⌊⋅⌋ represents rounding down.

In order to avoid invalid window functions, three constraints are given by [6]:

(6) I(0)=1

(7) $$W(f_{0}/2)\geq 0$$

(8) $$W(0)\geq W(f_{0}/2)$$

The above three constraints can be simplified as:

(9) $$0\leq \omega_{1}\leq \frac{1}{2\left[sinc\left(\left\lfloor \frac{f_{s}}{f_{0}}\right\rfloor \cdot w_{s}\right)-\cos\left(\pi \left\lfloor \frac{f_{s}}{f_{0}}\right\rfloor w_{s}\right)\right]}$$

(10) $$a=1-2\omega_{1}\cdot sinc\left(w_{s}\left\lfloor \frac{f_{s}}{f_{0}}\right\rfloor \right)$$

The SVA algorithm for non-integer Nyquist sampling rate may be seen as a filter with 3-point convolver. Define *g*(*m*) to be either the real or imaginary component of the image, and *m* is an index number running over the image pixel. So the SVA-filtered image can be expressed as:

(11) $$g^{\prime }(m)=ag(m)+\omega_{1}\left[g\left(m-\left\lfloor \frac{f_{s}}{f_{0}}\right\rfloor \right)+g\left(m+\left\lfloor \frac{f_{s}}{f_{0}}\right\rfloor \right)\right]$$

In order to get the value of *g′*(*m*), the following steps are implemented independently on both the real and imaginary parts of the image [4].

1. Calculate the value of *g′*(*m*) for *ω*_1_ = 0 and *ω*_1_ = *ω*_1_max_ (the upper limit of Equation (9)).
2. If the two values of *g′*(*m*) are opposite in sign, the output equals to zero at pixel m.
3. Otherwise, the output equals to the lowest magnitude.

**Figure 1:**
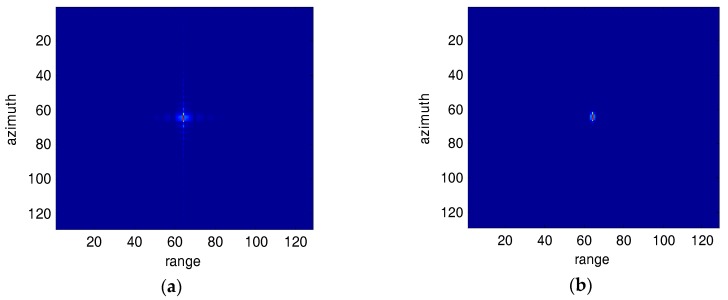
Impulse response for broadside SAR images (**a**) Original image; (**b**) SVA processed image.

**Figure 2:**
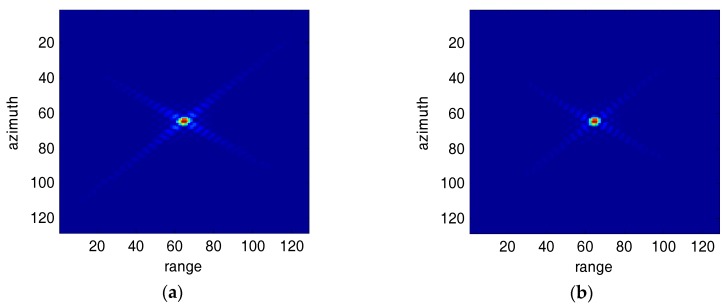
Impulse response for Squint SAR images (40°). (**a**) Original image; (**b**) SVA processed image.

Figure 1 and Figure 2 show a point target before and after SVA processed images for broadside SAR and squint SAR, respectively. The broadside SAR image’s sidelobes are suppressed efficiently as shown in Figure 1b, but the squint SAR image’s sidelobes are barely suppressed.

## 4. Two-Dimensional Sidelobe Correction Using D-SVA for Squint SAR Images

### 4.1. D-SVA for Non-Integer Nyquist Sampled Imagery

This section introduces a double-SVA (D-SVA) algorithm for non-integer Nyquist sampling rate, which has much lower sidelobe level than SVA and costs less computational complexity as well as time than 5-taps filter algorithm.

#### 4.1.1. Additional SVA Algorithm

From the above section, it is observed that Equation (5) is a special form of Equation (4). If the sampling points take the integers that are rounded up, it is given by:

(12) $$I(m)=a\cdot sinc(w_{s}m)+\omega_{1}\{sinc\left[w_{s}\left(m-\left\lceil \frac{f_{s}}{f_{0}}\right\rceil \right)\right]+sinc\left[w_{s}\left(m+\left\lceil \frac{f_{s}}{f_{0}}\right\rceil \right)\right]\}$$

where ⌈·⌉ means rounding up.

Similarly, Equations (9)–(11) can be rewritten as:

(13) $$0\leq \omega_{1}^{\prime }\leq \left|1/\left\{2\left[sinc\left(\left\lceil \frac{f_{s}}{f_{0}}\right\rceil \cdot w_{s}\right)-\cos\left(\pi \left\lceil \frac{f_{s}}{f_{0}}\right\rceil w_{s}\right)\right]\right\}\right|$$

(14) $$a^{\prime }=1-2\omega_{1}^{\prime }sinc\left(w_{s}\left\lceil \frac{f_{s}}{f_{0}}\right\rceil \right)$$

The new output signal in image domain can be expressed as:

(15) $$g^{\prime \prime }(m)=a^{\prime }g(m)+\omega_{1}^{\prime }\left[g\left(m-\left\lceil \frac{f_{s}}{f_{0}}\right\rceil \right)+g\left(m+\left\lceil \frac{f_{s}}{f_{0}}\right\rceil \right)\right]$$

*g″*(*m*) can be derived by executing the following steps:

1. Calculate the value of *g″*(*m*) for *ω′*_1_ = 0 and *ω′*_1_ = *ω′*_1_max_ (the upper limit of Equation (13)).
2. If the two values of *g″*(*m*) are opposite in sign, the output equals to zero at pixel m.
3. Otherwise, the output equals to the lowest magnitude.

**Figure 3:**
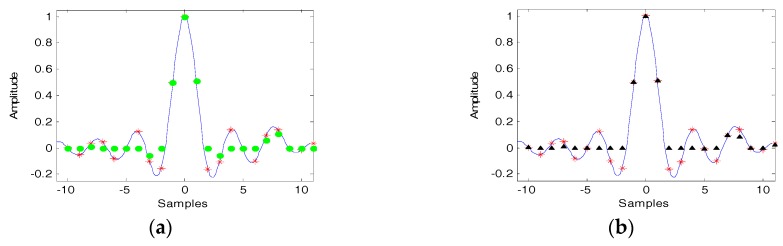
SVA and Additional SVA processed results ($w_{s}=0.6$). (**a**) SVA; (**b**) Additional SVA.

As shown in Figure 3, the over sampled signal is a sinc function in continuous time domain (blue line), and the red asterisks are the original sampling points. The green dots in Figure 3a are SVA processed result and the black triangles in Figure 3b are additional SVA processed result. It can be observed that Figure 3a,b have the same sampling amplitude of the mainlobe, but the sampled amplitudes of their sidelobes are different.

#### 4.1.2. D-SVA Algorithm

The outputs of D-SVA are the results selected from the SVA and additional SVA. By comparing the SVA processed result *g′*(*m*) and the additional SVA processed result $g^{\prime \prime }(m)$ at any pixel m, if either *g′*(*m*) or *g″*(*m*) is zero, the pixel equals to zero; otherwise, the pixel takes the minimum value.

**Figure 4:**
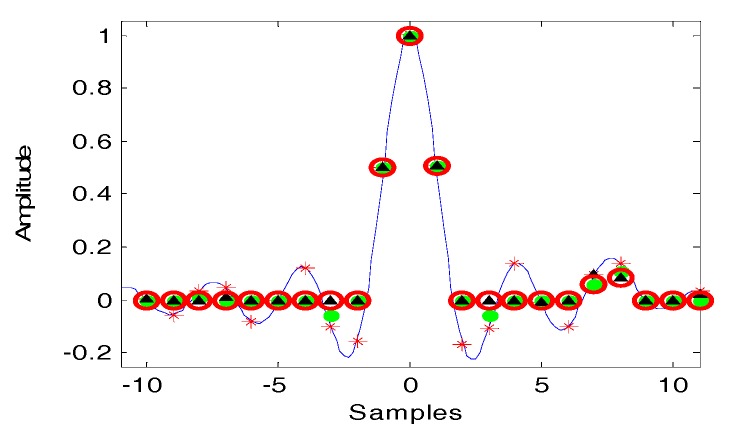
Comparison of different processing algorithms ($w_{s}=0.6$).

Figure 4 shows the D-SVA processed result compared to other algorithms. In Figure 4, the red asterisks are the original sampling points, the green dots are the SVA processed result, the black triangles are the additional SVA processed result, and the red circles are the final outputs using D-SVA for corresponding pixels.

### 4.2. Specturm Correction for Squint SAR Images

At present, various SVA algorithms have been applied to broadside SAR images [3,4,5,6,7,8,9]. But for squint SAR images, there are only a few papers proposed for sidelobe reduction [9]. When SAR is in squint mode, its antenna is not perpendicular to the flight path. The range and azimuth sidelobes are no longer parallel to the image principal axes (rows and columns). As shown in Figure 2, the impulse response for a point target has poor results after SAR signal processing and imaging processing (RMA). Therefore, the traditional SVA algorithms could hardly get all the sidelobe information, and the sidelobe level is still unbearable.

It should be noted that the support region for squint SAR images in 2-D frequency domain is not the same as the broadside SAR. This results in rotated sidelobe directions for the point targets that are not parallel to the principal axes. By applying the phase shift term, the squint SAR’s spectral shape can be transformed similarly to the broadside SAR whose range and azimuth sidelobes are vertical.

The Fourier transform pair can be written as:

(16) $$G(\xi )=\int_{-\infty }^{\infty }g(x)\exp\left\{-j2\pi \xi x\right\}dx$$

(17) $$g(x)=\int_{-\infty }^{\infty }G(\xi )\exp\left\{j2\pi \xi x\right\}d\xi$$

Their two-dimensional expressions are given by:

(18) $$G(\xi ,\eta )=\int_{-\infty }^{\infty }\int_{-\infty }^{\infty }g(x,y)\exp\left\{-j2\pi \left(\xi x+\eta y\right)\right\}dxdy$$

(19) $$g(x,y)=\int_{-\infty }^{\infty }\int_{-\infty }^{\infty }G(\xi ,\eta )\exp\left\{+j2\pi \left(\xi x+\eta y\right)\right\}d\xi d\eta$$

According the Fourier transform property, the following two equations are given by:

(20) $$H_{FT}(\xi ,y)=\int_{-\infty }^{\infty }g(x,y)\exp\left\{-j2\pi \xi x\right\}dx$$

(21) $$H_{TF}(x,\eta )=\int_{-\infty }^{\infty }g(x,y)\exp\left\{-j2\pi \eta y\right\}dy$$

The definition of time shifting is given by:

(22) g(x)↔G(ξ)

(23) $$g(x-x_{0})↔G(\xi )\exp\left\{-j2\pi \xi x_{0}\right\}$$

The effect of a time shift on a signal is to introduce a phase shift $-2\pi ft_{0}$ in its transform.

**Figure 5:**
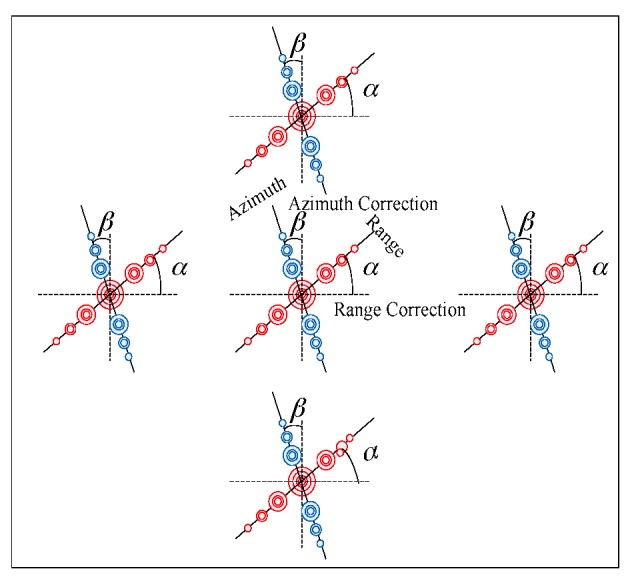
The schematic diagram of range and azimuth sidelobes for squint SAR image.

SAR image data is recorded as *g*(*x,y*), in which *x* and *y* represent the azimuth and range of the image, respectively. As shown in Figure 5, define ***α*** as the range sidelobe correction angle between the original range sidelobe and the corrected range sidelobe, and the corresponding time delay in time domain as *k*_1_*y*:

(24) $$H_{FT}(\xi ,y)\cdot \exp\left\{j2\pi \xi k_{1}y\right\}\overset{RFT}{↔}G(\xi ,\eta -k_{1}\xi )$$

Similarly, in Figure 5, define the azimuth sidelobe correction angle as *β*, and the corresponding time delay is *k*_2_*x*:

(25) $$H_{TF}(x,\eta )\cdot \exp\left\{j2\pi \eta k_{2}x\right\}\overset{AFT}{↔}G(\xi -k_{2}\eta ,\eta )$$

The squint SAR spectrum is:

(26) $$g(x+k_{1}y,y+k_{2}x)\overset{2DFT}{↔}G(\xi -k_{2}\eta ,\eta -k_{1}\xi )$$

when $k_{2}\neq 0$ and $k_{1}\neq 0$, from Equations (24) and (26):

(27) $$G(\xi -k_{2}\eta ,\eta -k_{1}\xi )\overset{RIFT}{↔}H_{FT}(\xi -k_{2}\eta ,y)\cdot \exp\left\{j2\pi \xi k_{1}y\right\}$$

Therefore, the range corrected spectrum is given by:

(28) $$H_{FT}(\xi -k_{2}\eta ,y)\cdot \exp\left\{j2\pi \xi k_{1}y\right\}\cdot \exp\left\{-j2\pi \xi k_{1}^{'}y\right\}=H_{FT}\left(\xi -k_{2}\eta ,y\right)\exp\left\{-j2\pi \xi \left(k_{1}^{'}-k_{1}\right)y\right\}$$

where $\exp\left\{-j2\pi \xi k_{1}^{\prime }y\right\}$ is the phase shift term determined by the range sidelobe correction angle, $k_{1}^{\prime }=tan\alpha^{\prime }$, and *α*′ is the estimate angle for range sidelobes. When $k_{1}^{\prime }=k_{1}$, the range sidelobes are corrected after azimuth Inverse Fourier Transform (IFT).

The azimuth sidelobes are corrected after the range sidelobe correction, and the expression is given by:

(29) $$g(x,y+k_{2}x)\overset{2DFT}{↔}G(\xi -k_{2}\eta ,\eta )$$

From Equations (25) and (27), the following transformation holds

(30) $$G(\xi -k_{2}\eta ,\eta )\overset{AIFT}{↔}H_{TF}(x,\eta )\cdot \exp\left\{j2\pi \eta k_{2}x\right\}$$

Therefore, the azimuth corrected spectrum is given by:

(31) $$H_{TF}(x,\eta )\cdot \exp\left\{j2\pi \eta k_{2}x\right\}\cdot \exp\left\{-j2\pi \eta k_{2}^{\prime }x\right\}=H_{TF}\left(x,\eta \right)\cdot \exp\left\{-j2\pi \eta \left(k_{2}^{\prime }-k_{2}\right)x\right\}$$

where $\exp\left\{-j2\pi \eta k_{2}^{\prime }x\right\}$ is the phase shift term determined by the azimuth sidelobe correction angle, $k_{1}^{\prime }=tan\beta^{\prime }$, and *β*′ is the estimate angle for azimuth sidelobes. When $k_{2}^{\prime }=k_{2}$, the azimuth sidelobes are corrected after range IFT.

According to the time shifting property, the squint SAR image can be corrected as the broadside SAR image. The corrected data can then accomplish sidelobe suppression by applying the D-SVA algorithm. Since the range and azimuth sidelobes are both changed after correction, while the space geometrical relation of the SAR image is also changed [16], it is necessary to recover the original space geometrical location of the image after the D-SVA processing. Thanks to the time shifting property, this can be achieved because the range and azimuth direction corrections are invertible.

**Figure 6:**
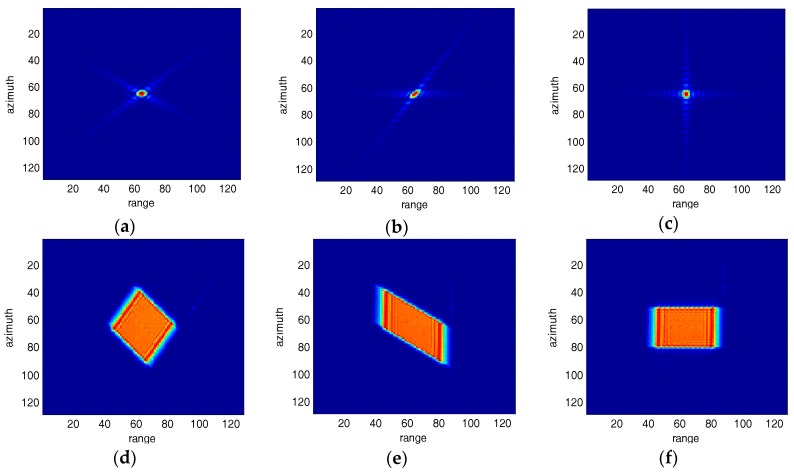
The squint SAR images (**a**) Squint angle 40° SAR image; (**b**)Range sidelobes corrected; (**c**)Range and azimuth sidelobes corrected; (**d**~**f**) are the 2-D spectrum images for (**a**~**c**), respectively.

As shown in Figure 6, Figure 6a is a point target for 40° squint SAR image; Figure 6b is the processed image for Figure 6a after range sidelobe correction; Figure 6c is the processed image for Figure 6a after range and azimuth sidelobe correction; (d~f) are the corresponding 2-D spectrum images for (a~c), respectively.

### 4.3. Sidelobe Reduction for Squint SAR Images

Sidelobe reduction for squint SAR images includes the following steps.

1. First, transform the squint SAR images *E*_0_ into frequency domain and shift the center of the frequency spectrum to the data’s center. Then by applying an inverse IFT to the data in time domain, the new data *E*_1_ is recorded.
2. Range sidelobe correction

Apply FT to the azimuth of *E*_1_. According to the sidelobe directions of the strong scatterer, estimate the range sidelobe angle *α_y_*, so:

(32) $$dy_{ri}=dr_{i}\cdot \tan(\alpha_{y})$$

(33) $$dY_{r}=\left[dy_{r1},dy_{r2},\cdots ,dy_{rN_{r}}\right]$$

(34) $$E_{2}=ift_{A}\left[ft_{A}\left(E_{1}\right)\cdot \exp\left\{-j2\pi \cdot \xi \cdot dY_{r}\right\}\right]$$

where $dr_{i}=\left(\frac{N_{r}}{2}-i\right),\;i=1,2,L,N_{r}$, *ξ* is a column vector, $\xi ={\left[-0.5:1:\left(0.5-\frac{1}{N_{a}}\right)\right]}^{T}$, *ft_A_*(·) and *ift_A_*(·) are the azimuth FT and azimuth IFT, respectively.

3. Azimuth sidelobe correction

Apply FT to the range of *E*_2_. According to the sidelobe directions of the strong scatterer, estimate the azimuth sidelobe angle *α_x_*, so:

(35) $$dx_{aj}=da_{j}\cdot \tan(\alpha_{x})$$

(36) $$dX_{a}={\left[dx_{a1},dx_{a2},\cdots ,dx_{aN_{a}}\right]}^{T}$$

(37) $$E_{3}=ift_{R}\left[ft_{R}(E_{2})\cdot \exp\left\{-j2\pi \cdot dX_{a}\cdot \eta \right\}\right]$$

where $da_{j}=\left(\frac{N_{a}}{2}-j\right),\;j=1,2,L,N_{a}$ , η is a row vector , $\eta =\left[-0.5:1:\left(0.5-\frac{1}{N_{r}}\right)\right]$, *ft_R_*(·) and *ift_R_*(·) are the range FT and range IFT, respectively.

4. Use D-SVA to process the range and azimuth, obtaining *E*_4_.
5. Use azimuth phase shift term to rotate the azimuth sidelobes for *E*_4_:
  (38) $$E_{5}=ift_{R}\left[ft_{R}(E_{4})\cdot \exp\left\{j2\pi \cdot dX_{a}\cdot \eta \right\}\right]$$
6. Use range phase shift term to rotate the range sidelobes for *E*_5_:
  (39) $$E_{6}=ift_{A}\left[ft_{A}\left(E_{5}\right)\cdot \exp\left\{j2\pi \cdot \xi \cdot dY_{r}\right\}\right]$$
7. At each spatial location, select the minimum value between *E*_6_ and *E*_1_ as output.

**Figure 7:**
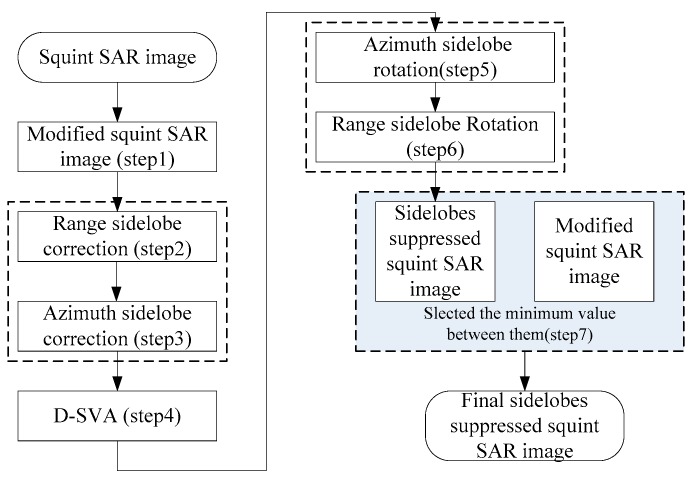
The flowchart of sidelobe reduction algorithm for squint SAR images.

To summarize, Figure 7 is the flowchart of the sidelobe reduction algorithm for squint SAR images.

## 5. Simulation Results and Analysis

**Figure 8:**
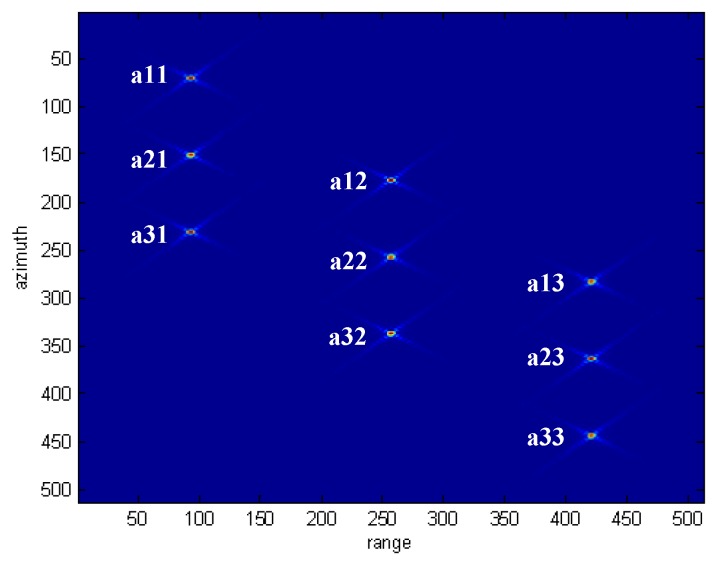
The original squint SAR Image.

Table 1 presents the simulation parameters for a squint SAR system. Figure 8 shows the original 40° squint SAR image, which has nine point targets. The sidelobes of the nine targets are seen clearly in Figure 8.

**Figure 9:**
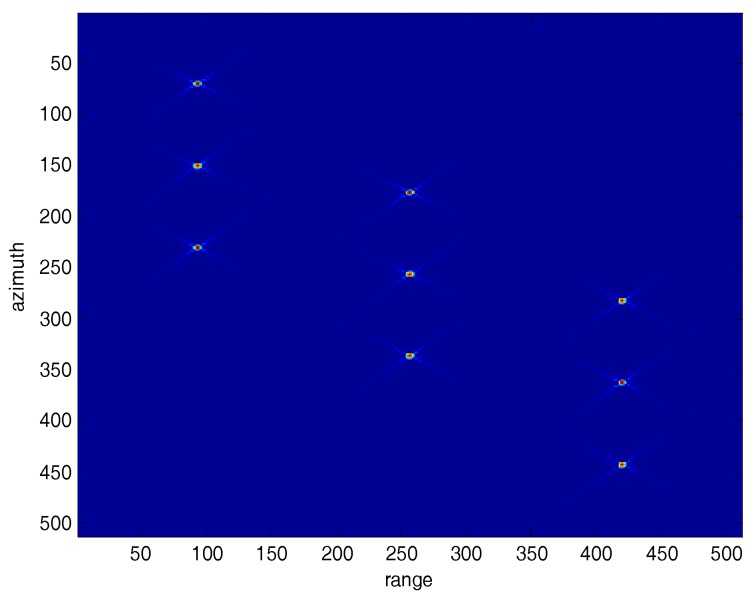
The squint SAR processed image using the traditional SVA algorithm.

**Figure 10:**
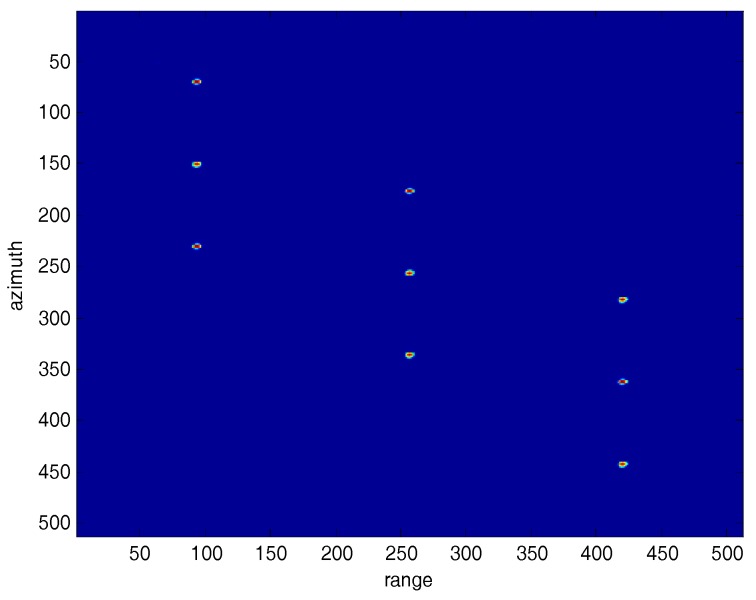
The squint SAR processed image using the proposed framework.

Figure 9 and Figure 10 compare the processed images after the traditional SVA squint SAR image processing and the proposed algorithm. In Figure 9, the nine point targets’ sidelobes dim slightly but are still observable, which means that the sidelobe energy is suppressed to some extent but not all by using traditional SVA algorithm. In comparison, Figure 10 indicates that the squint SAR image sidelobes are suppressed effectively by using the proposed framework. 

**Figure 11:**
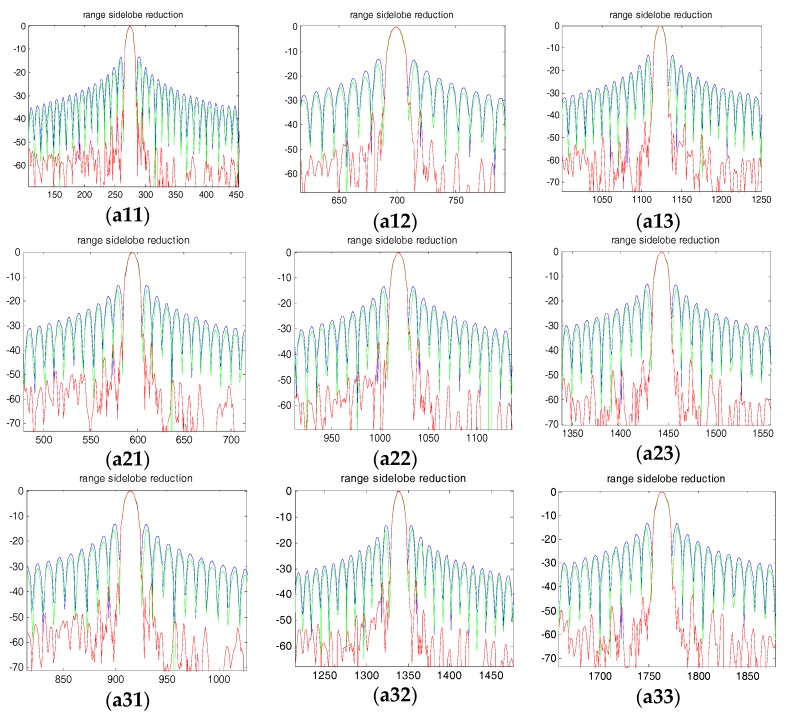
Range profiles for the nine points. (**a11**~**a33**) are the nine points’ range profiles, respectively.

**Figure 12:**
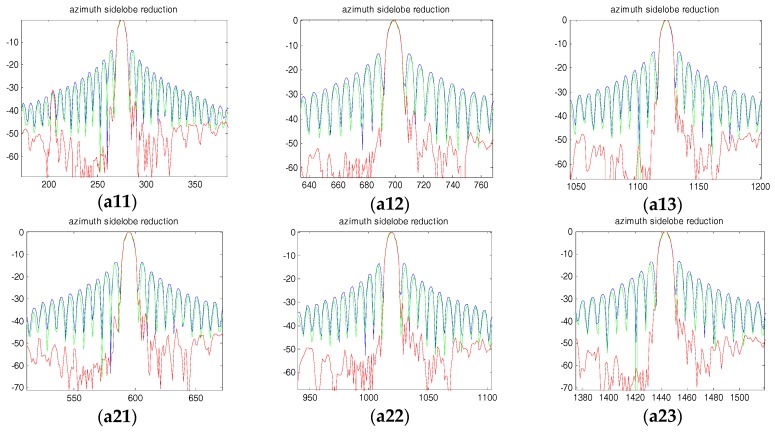

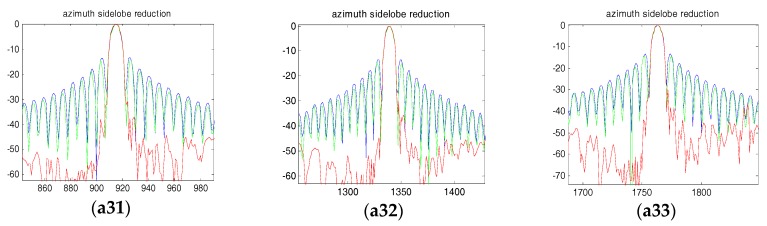
Azimuth profiles for the nine points. (**a11**~**a33**) are the nine points’ azimuth profiles, respectively.

Figure 11 and Figure 12 show the range and azimuth profiles of the nine point targets. The blue line corresponds to the original result of the squint SAR image (Figure 8), the green line is the result processed by the traditional SVA (Figure 9), and the red line is the result processed by the proposed framework (Figure 10). It is apparent that the traditional SVA algorithm is ineffective for squint SAR image sidelobe reduction while the proposed framework can suppress sidelobes well and keep the resolution at the same time.

Table 2 presents the sidelobe reduction results for the nine point targets. It indicates that the traditional SVA algorithm is ineffective for the squint SAR image sidelobe reduction, in which the peak sidelobe ratio (PSLR) of the nine point targets’ range and azimuth only decreased slightly. On the other hand, the nine point targets’ PSLR all fell below −30 dB by using the proposed framework.

**Figure 13:**
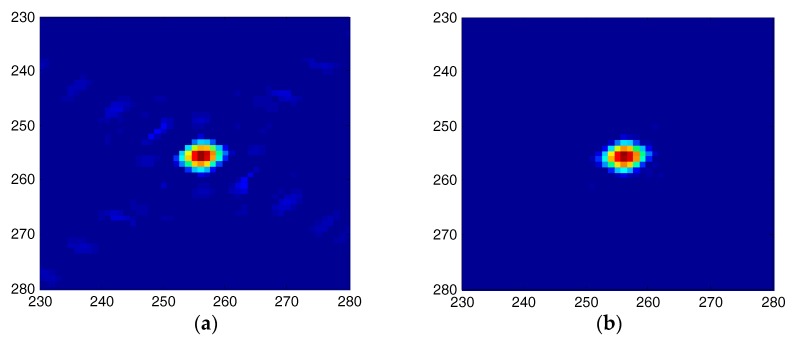
Sidelobe reduction results of squint SAR image (**a**) Processed result by using the method of References [9]; (**b**) Processed result by using the proposed method.

**Figure 14:**
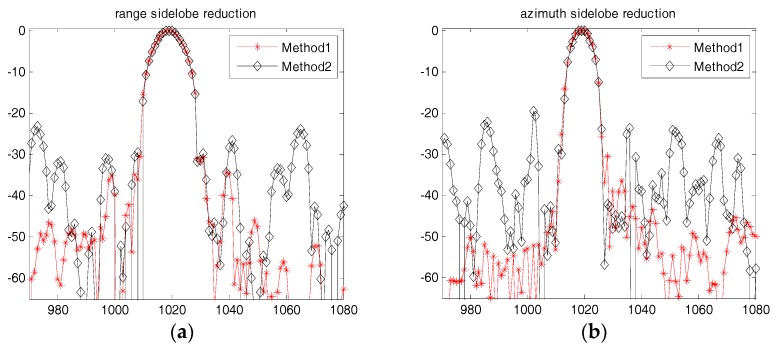
Range and azimuth profiles comparison between the two methods (**a**) Range profiles; (**b**) Azimuth profiles.

The proposed algorithm is further compared with the method in reference [9]. Figure 13 presents the sidelobe reduction results of the center point (a22) as shown in Figure 8. Figure 13a is the processed result by using the method of Reference [9]. From this figure, it can be observed that the point target’s sidelobes are suppressed to some extent, but they are still traceable in the processed image. Figure 13b is the processed result by using the proposed method. The point target’s sidelobes are suppressed very well and can hardly be seen in this figure. Figure 14 is the comparison results for the range and azimuth profiles between the two algorithms, in which Figure 14a presents the range profiles, and Figure 14b presents the azimuth profiles. In Figure 14, the line with red asterisks is processed by our proposed method (method 1) and the line with black triangles is processed by the method of [9] (method 2). As shown in Figure 14, it indicates that our proposed method has a better sidelobe reduction result for both the range and azimuth directions. In addition, it should be noted that the method of [9] costs much more time by using 5-taps SVA method.

**Figure 15:**
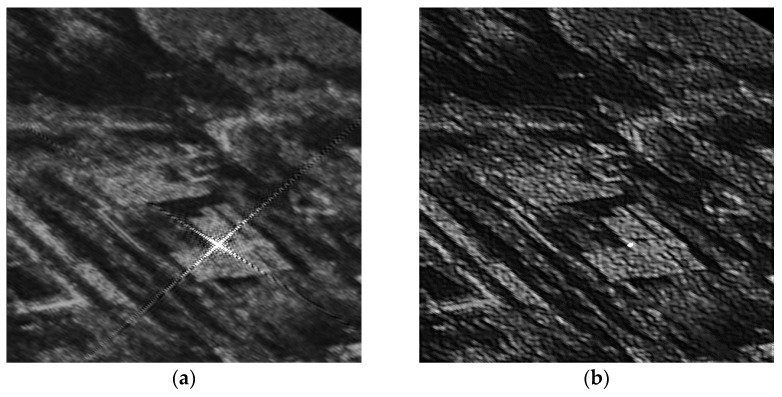
The unprocessed and processed squint SAR images (**a**) Unprocessed image; (**b**) Processed image by using our proposed method.

Figure 15 further illustrates an unprocessed and processed squint SAR image for a real scene. Figure 15a is the unprocessed image and Figure 15b is the processed image. This image shows a farm near Tianjin with a corner reflector in the center of the test scene. It’s a C-band SAR system, whose resolution is 3 m and the equivalent squint angle is 40°. As backscatter intensity of the corner reflector far exceeds the background image, the sidelobes of the corner reflector in the image appears obviously. After D-SVA algorithm processing, the sidelobes of the corner reflector has been significantly suppressed. The corner reflector is common calibration equipment for SAR system. The result based on real scene can effectively verify the experimental conclusion of this paper.

## 6. Conclusions

A novel two-dimensional sidelobe suppression algorithm using D-SVA is proposed for squint SAR images. In this paper, the sidelobe distribution of squint SAR image is approximately corrected to broadside SAR image by introducing the shift phase term, and the D-SVA algorithm can accurately process the sidelobe energy of any squints angle. This algorithm’s computational efficiency is twice as fast as SVA. It can reduce the sidelobe energy efficiently without degrading resolution. This algorithm is independent of imaging algorithm and is capable to reduce sidelobes for all squint SAR images. It simplifies the sidelobe reduction process by skipping frequency domain interpolation and sampling parameters recalculation. The D-SVA algorithm can also be applied directly to broadside SAR images for sidelobe reduction, which can achieve better sidelobe suppression results than the SVA algorithm.

## Figures and Tables

**Table 1:** Simulation parameters.

Parameters	Value	Units
Wavelength	0.05657	m
Pulse bandwidth	100	MHz
Sampling rate	120	MHz
Velocity	250	m/s
Pulse duration	1	μs
Pulse repetition frequency	500	Hz
Squint angle	40	deg
Synthetic aperture time	2.5136	sec
Central slant range	22.057	Km

**Table 2:** Sidelobe reduction results for the nine point targets.

Performance Index		Traditional SVA	Proposed Framework
a11	Range PSLR	−15.38 dB	−33.13 dB
	Azimuth PSLR	−14.42 dB	−30.63 dB
a12	Range PSLR	−14.73 dB	−30.64 dB
	Azimuth PSLR	−14.67 dB	−30.51 dB
a13	Range PSLR	−14.95 dB	−39.42 dB
	Azimuth PSLR	−14.54 dB	−30.88 dB
a21	Range PSLR	−15.36 dB	−33.36 dB
	Azimuth PSLR	−14.42 dB	−30.64 dB
a22	Range PSLR	−14.72 dB	−30.80 dB
	Azimuth PSLR	−14.63 dB	−30.44 dB
a23	Range PSLR	−15.04 dB	−39.62 dB
	Azimuth PSLR	−14.59 dB	30.43 dB
a31	Range PSLR	−15.33 dB	−33.58 dB
	Azimuth PSLR	−14.43 dB	−30.84 dB
a32	Range PSLR	−14.75 dB	−30.85 dB
	Azimuth PSLR	−14.61 dB	−30.26 dB
a33	Range PSLR	−15.07 dB	−41.15 dB
	Azimuth PSLR	−14.59 dB	−30.34 dB
